# Three novel marine species of the genus *Reichenbachiella* exhibiting degradation of complex polysaccharides

**DOI:** 10.3389/fmicb.2023.1265676

**Published:** 2023-12-14

**Authors:** Neak Muhammad, Forbes Avila, Olga I. Nedashkovskaya, Song-Gun Kim

**Affiliations:** ^1^Biological Resource Center/Korean Collection for Type Cultures (KCTC), Korea Research Institute of Bioscience and Biotechnology, Daejeon, Republic of Korea; ^2^Department of Environmental Biotechnology, KRIBB School of Biotechnology, University of Science and Technology (UST), Daejeon, Republic of Korea; ^3^G.B. Elyakov Pacific Institute of Bioorganic Chemistry of the Far-Eastern Branch of the Russian Academy of Sciences, Vladivostok, Russia

**Keywords:** *Bacteroidota*, genome analyses, polysaccharide degradation, carbohydrate-active enzymes, CRISPRCas

## Abstract

Three novel strains designated ABR2-5^T^, BKB1-1^T^, and WSW4-B4^T^ belonging to the genus *Reichenbachiella* of the phylum *Bacteroidota* were isolated from algae and mud samples collected in the West Sea, Korea. All three strains were enriched for genes encoding up to 216 carbohydrate-active enzymes (CAZymes), which participate in the degradation of agar, alginate, carrageenan, laminarin, and starch. The 16S rRNA sequence similarities among the three novel isolates were 94.0%–94.7%, and against all three existing species in the genus *Reichenbachiella* they were 93.6%–97.2%. The genome sizes of the strains ABR2-5^T^, BKB1-1^T^, and WSW4-B4^T^ were 5.5, 4.4, and 5.0 Mb, respectively, and the GC content ranged from 41.1%–42.0%. The average nucleotide identity and the digital DNA–DNA hybridization values of each novel strain within the isolates and all existing species in the genus *Reichenbachiella* were in a range of 69.2%–75.5% and 17.7–18.9%, respectively, supporting the creation of three new species. The three novel strains exhibited a distinctive fatty acid profile characterized by elevated levels of iso-C_15:0_ (37.7%–47.4%) and C_16:1_ ω5c (14.4%–22.9%). Specifically, strain ABR2-5^T^ displayed an additional higher proportion of C_16:0_ (13.0%). The polar lipids were phosphatidylethanolamine, unidentified lipids, aminolipids, and glycolipids. Menaquinone-7 was identified as the respiratory quinone of the isolates. A comparative genome analysis was performed using the KEGG, RAST, antiSMASH, CRISPRCasFinder, dbCAN, and dbCAN-PUL servers and CRISPRcasIdentifier software. The results revealed that the isolates harbored many key genes involved in central metabolism for the synthesis of essential amino acids and vitamins, hydrolytic enzymes, carotenoid pigments, and antimicrobial compounds. The KEGG analysis showed that the three isolates possessed a complete pathway of dissimilatory nitrate reduction to ammonium (DNRA), which is involved in the conservation of bioavailable nitrogen within the ecosystem. Moreover, all the strains possessed genes that participated in the metabolism of heavy metals, including arsenic, copper, cobalt, ferrous, and manganese. All three isolated strains contain the class 2 type II subtype C1 CRISPR-Cas system in their genomes. The distinguished phenotypic, chemotaxonomic, and genomic characteristics led us to propose that the three strains represent three novel species in the genus *Reichenbachiella*: *R. ulva*e sp. nov. (ABR2-5^T^ = KCTC 82990^T^ = JCM 35839^T^), *R. agarivorans* sp. nov. (BKB1-1^T^ = KCTC 82964^T^ = JCM 35840^T^), and *R. carrageenanivorans* sp. nov. (WSW4-B4^T^ = KCTC 82706^T^ = JCM 35841^T^).

## 1 Introduction

The marine ecosystem is one of the largest and most intricate aquatic systems on the planet. The marine microbes participate in the maintenance and regulation of the biogeochemical cycles of the sea (Falkowski et al., 2008; Murillo et al., 2019). Marine microbes degrade dead plants, animals, and algae and turn them into useful nutrients that can allow further growth of these organisms. Furthermore, the genomes of these microbes are highly enriched with genes for the breakdown of complex molecules such as pollutants, peptides, and polysaccharides (Gao et al., 2017; McKee et al., 2021).

There are very different types of polysaccharides, such as agar, alginate, chitin, carrageenan, cellulose, fucoidans, laminarin, pectin, porphyrin, ulvane, and xylan (Helbert, 2017). Polysaccharides are different based on their isolation sources, chemical composition, and structure. Agar, alginate, carrageenan, fucoidan, laminarin, and ulvan originate mainly from diverse algae and phytoplankton, while cellulose, pectin, and xylan are derived from plants, and the remaining are from animals and fungi (de Jesus Raposo et al., 2015; Helbert, 2017). Among them, carrageenan, fucoidan, and ulvan, are classified as sulfated polysaccharides (Bhuyan et al., 2023). Polysaccharides are made of sugar monomers connected through glycosidic linkages, including but not limited to β-1,3, β-1,4, β-1,6, α-1,3, and α-1,4 linkages (Bäumgen et al., 2021). The cell walls of macroalgae and phytoplankton serve as readily accessible sources of these polysaccharides (Ehrlich et al., 2018; Shao and Duan, 2022). Macroalgae consist of three main groups, such as red, brown, and green, which are vital parts of the marine food chain (Øverland et al., 2019). Furthermore, macroalgae provide a solid attachment for many groups of marine bacteria to form a mutually benefit interaction between macroalgae and bacteria or for bacteria to invade macroalgae and breakdown the complex polysaccharides of macroalgae (Singh and Reddy, 2014; Brunet et al., 2022). Numerous groups of algal polysaccharide-degrading bacteria have been isolated in marine environments, contributing to algal biomass recycling and the carbon cycle (Martin et al., 2015). The oligosaccharides produced from degradation of the polysaccharides have been reported to exhibit various biological activities, making them applicable in the functional food, cosmetic, and medical sectors (de Jesus Raposo et al., 2015; Ruocco et al., 2016). For example, carrageenan and alginate oligosaccharides show a wide range of biological activities, including antiviral, anticancer, antioxidant, anti-angiogenic, immunomodulation, antimicrobial, antihypertensive, and antidiabetic activities (Liu et al., 2019; Zhao et al., 2021). Consequently, there is a strong demand to explore novel bacterial strains that can effectively degrade complex polysaccharides. The microbial degradation of polysaccharides for the production of oligosaccharides offers significant advantages over chemical methods. Microbial degradation procedures are environmentally friendly, highly specific, biodegradable, and cost and energy-efficient (Deng et al., 2023).

The marine microbes are enriched with genes encoding enzymes that potentially participate in polysaccharide degradation, known as carbohydrate-active enzymes (CAZymes; McKee et al., 2021). In the bacterial genome, these CAZymes, transporters and regulator proteins are organized into a system called polysaccharide utilization loci (PUL; Terrapon et al., 2015). Currently, there are approximately 300 CAZymes protein families that categorized into five classes: glycoside hydrolases (GHs), glycosyltransferases (GTs), polysaccharide lyases (PLs), carbohydrate esterases (CEs), and carbohydrate-binding modules (CBMs; Cantarel et al., 2009). There are numerous strains belonging to the phylum *Bacteroidota* that are highly enriched with CAZymes and have the potential to degrade diverse types of polysaccharide (Hehemann et al., 2012; Mann et al., 2013; McKee et al., 2021).

The phylum *Bacteroidota*, formerly recognized as *Bacteroidetes*, is considered as a major group of marine heterotrophic bacterioplankton (Paster et al., 1994). At the time of writing, the phylum *Bacteroidota*^1^ comprises six classes and six orders. The class *Cytophagia* consist of single order called *Cytophagales*^2^ which mainly composed of 20 families. Among these, the family *Reichenbachiellaceae* comprises only three validly published genera.^3^ The genus *Reichenbachia* was originally proposed by Nedashkovskaya in 2003 in the family *Flammeovirgaceae* (Nedashkovskaya et al., 2003). The name *Reichenbachia* was subsequently changed to *Reichenbachiella* and separated into the family *Reichenbachiellaceae* (Nedashkovskaya et al., 2005; García-López et al., 2019). Over the past few years, a total of three *Reichenbachiella* species have been described,^4^ namely, *R. agariperforans* (Nedashkovskaya et al., 2005) *R. faecimaris* (Cha et al., 2011), and *R. versicolor* (Shi et al., 2018). They have been isolated from sea coelenterate, tidal-flat sediment, and red algae, respectively. The species of genus *Reichenbachiella* are characterized as heterotrophic, Gram-stain-negative, aerobe, non-motile, non-spore producer, rod-shaped bacteria. The menaquinone-7 (MK-7) is common among the species of genus *Reichenbachiella* (Shi et al., 2018). Menaquinone-7 (MK-7) is a type of quinone molecule which is essential cofactor in electron transport chains and plays crucial roles in bacterial physiology and metabolism Menaquinone can also be used as a chemotaxonomic marker in the field of microbial taxonomy (Hiraishi, 1999). To date, relative few studies on the comprehensive genome analysis for applications of the genus *Reichenbachiella* are available.

While investigating the microbial diversity of the tidal flats in the West Sea, Korea, a high number of novel bacteria capable of degrading complex polysaccharides were isolated (Muhammad et al., 2023; Nguyen et al., 2023). Three novel strains, ABR2-5^T^, BKB1-1^T^, and WSW4-B4^T^, were isolated from macroalgae and sea mud. On the basis of a taxonomic study using a polyphasic approach, we propose that these three strains should be included in the genus *Reichenbachiella* as representatives of three novel species. Furthermore, we report the polysaccharide-degrading abilities of these three isolates. The strains possess the ability to degrade various complex polysaccharides of agar, alginate, carrageenan, laminarin, and starch and carry a high number of genes for CAZymes in their genomes. Moreover, the strains carry genes for the production of secondary metabolites, the synthesis of essential amino acids and vitamins, class 2 type II subtype C1 CRISPR-Cas system, heavy metal metabolism, and important pathways that participate in the nitrogen cycle of coastal ecosystems.

## 2 Materials and methods

### 2.1 Isolation and identification of gliding bacterial strains

Three samples were collected from different locations in the West Sea, Korea. In late autumn 2022, 100 gram red alga *Chondrus* sp. was collected from a beach in Byeonsan (35° 40′ 53.76″ N 126° 31′ 51.96″ E). One hundred gram green alga *Ulva* sp. was collected from Aphae Island (34° 49′ 52.8″ N 126° 22′ 48.5″ E) on June 2021. Fifty gram sea mud was collected from an estuary at Bigeum Island (34° 41′ 23.3″ N 125° 55′ 13.4″ E) on June 2021. The specimens were taken to the lab immediately and processed.

To isolate gliding bacteria, we used low-nutrient media composed of 60% (v/v) seawater, 1.5% (w/v) agar, and 50 mg/L cycloheximide. To isolate novel strains, we aimed to replicate natural conditions for the bacteria. Therefore, seawater from the same sampling area was collected and used in media preparation. The low-nutrient media, prepared with seawater, enabled us to isolate marine gliding bacteria. We used 60% (v/v) seawater for all media preparation instead of 100% because the samples were collected from tidal flats where salinity could be lower.

One gram of each algae was diced into small pieces and placed at the center of solid media. The sea mud sample was also placed in the same medium. All plates were incubated for 7 days at 15°C and then regularly observed for gliding bacteria using a stereo microscope (ZEISS Stemi 508; Nguyen et al., 2023). The gliding colonies at the margin were transferred with a sterile needle onto marine agar 2,216 (MA; BD) and modified VY/2 agar media [MVY; 60% (v/v) seawater, 5 g/L baker’s yeast (Sigma) and 25 mg/L filtered sterile vitamin B_12_] until pure cultures were obtained. A strain of a circular, smooth, orange-pigmented colony was designated as strain ABR2-5^T^, while a strain of an orange-pigmented colony that hydrolyzed agar was designated as strain BKB1-1^T^, and a strain of smooth circular pale-yellow colonies was designated as strain WSW4-B4^T^. Bacterial strains isolated were preserved in 20% glycerol at-80°C and in lyophilized ampoules at 4°C.

The genomic DNA, extracted from cells cultivated on MA, was used for the amplification of 16S rRNA genes using four universal primers (27F, 518F, 805R, and 1492R; Pheng et al., 2020). These primers are designed to determine a nearly complete sequence of the 16S rRNA gene. The complete sequences were then assembled by using Vector NTI software (Invitrogen). The 16S rRNA sequence were queried to search the similar sequences from the EzBioCloud server^5^ (Yoon et al., 2017a). The similar sequences downloaded from the EzBioCloud server were used to construct neighbor-joining (NJ; Saitou and Nei, 1987), maximum-likelihood (ML; Felsenstein, 1981), and maximum parsimony (MP) phylogenetic trees (Fitch, 1971) in Molecular Evolutionary Genetics Analysis (MEGA X) software (Kumar et al., 2018). The robustness of the sequence clustering was evaluated using the bootstrap resampling method with 1,000 replicates. *Flammeovirga aprica* NBRC 15941^T^ was incorporated into the analysis as an outgroup.

### 2.2 Phenotypic characterization

The colonies’ shapes were observed on MA plates after 3 days of cultivation. We used the BBL™ Gram Stain Kit (BD, USA) for Gram-staining. The shape and size of the bacterial strains was observed by a scanning electron microscope (Regulus 8,100, Hitachi; Jeon et al., 2022). Motility was observed by using a hanging-drop technique, and gliding activity was observed by growing the strain on low-nutrient media composed of sea water and 0.7% (w/v) agar (Tittsler and Sandholzer, 1936). The optimal temperature and salt tolerance for the growth was determined by using MA (Muhammad et al., 2022) while the optimal pH for the growth was tested in MB (Muhammad et al., 2023). To determine the growth under an anaerobic condition, all three strains were tested on a solid medium composed of 0.2% (w/v) glucose, 2% (w/v) NaCl, 0.1% (w/v) polypeptone, 0.1% (w/v) KH_2_PO_4,_ 0.1% (w/v) K_2_HPO_4_, 0.2% (w/v) NH_4_Cl, 0.14% (w/v) MgSO4, NaNO_3_ (w/v) 0.17, 1.5% (w/v) agar, 0.1% (w/v) resazurin, 0.05% (w/v) cysteine HCl, 1 mL trace elements, and 1 mL multivitamins (Wolin et al., 1963; Kim et al., 2001; Bae et al., 2020).

Catalase and oxidase activities were tested by using 3% (v/v) H_2_O_2_ and 1% (w/v) tetramethyl-*p*-phenylenediamine reagents, respectively (Ueno et al., 2021). The Cowan & Steel protocol was used to test the hydrolysis of Tweens 20, 40, 80, and casein (Phillips, 1993). The activities of DNase were determined using DNase agar (Difco; Jeffries et al., 1957). The 20% (w/v) KOH solution was used to assess the presence of flexirubin-type pigments (Lin et al., 2020). The enzymatic activities were tested using the API ZYM kit (bioMérieux), and the ability to utilize various carbon sources was assessed using the API 20E and API 50CH kits (bioMérieux), and GEN III Microplates (from Biolog; Jeong et al., 2020).

The cellular fatty acids, quinones, and polar lipids were determined for the three strains. For the fatty acid analysis, cells grown on MA of the same growth stage were collected. The fatty acid was extracted using a standard MIDI protocol (version 6.2; Sasser, 1990). For the determination of respiratory quinones, Komagata and Suzuki protocol was used (Komagata and Suzuki, 1988). For the analysis of polar lipids, Komagata and Suzuki method was used to extract the compound from the cell biomass using chloroform/methanol followed by two-dimensional TLC (Komagata and Suzuki, 1988). Finally, the TLC plates were dried and sprayed with 0.2% ninhydrin, α-naphthol, molybdenum blue, and 0.5% phosphomolybdic acid to detect amino lipids, glycollipids, phospholipids, and total lipids (Komagata and Suzuki, 1988).

### 2.3 Genome sequencing and analysis

The genomic DNA of the three strains ABR2-5^T^, BKB1-1^T^, and WSW4-B4^T^ was extracted using NucleoSpin Microbial DNA kit (Macherey-Nagel, Germany). The concentration and purity of the genomic DNA was determined using a NanoDrop spectrophotometer (ThermoScientific, United States), and the fragmentation of DNA was visualized by gel electrophoresis using 1% (w/v) agarose gel.

Oxford Nanopore Technologies (ONT, United Kingdom) platform was used for genome sequencing. Ligation sequencing kit (SQK-LSK112), native barcoding kit (SQK-NBD112.24), R10.4 FLO-MIN112 flow cells, and MinION device were used in the sequencing process. Basecalling was carried out with default parameters using MinKNOW software version 22.10.7 and Guppy 6.3.8 (Wick et al., 2019). *De novo* assembly was done by Flye version 2.9.1^6^ (Kolmogorov et al., 2019). To assess the completeness and contamination of the assembled genomes, we used CheckM version 1.2.2^7^ and Busco version 5.4.4^8^ (Parks et al., 2015; Manni et al., 2021).

### 2.4 Genome based phylogeny

To determine the taxonomic position of strains ABR2-5^T^, BKB1-1^T^, and WSW4-B4^T^, we calculated the average nucleotide identity (ANI) and digital DNA–DNA hybridization (dDDH) using EZBiocloud’s ANI calculator^9^ (Yoon et al., 2017b) and DSMZ’s Genome to Genome Distance Calculator version 3.0^10^ (Meier-Kolthoff et al., 2013). We generated the genomic phylogenetic tree using the 92 prokaryotic core-genes according to the up-to-date bacterial core gene (UBCG) pipeline (Na et al., 2018) with *Flammeovirga aprica* JL-4^T^ (GCF012844305) as an outgroup.

### 2.5 Genome functional analysis

First, the three genomes of strains ABR2-5^T^, BKB1-1^T^, and WSW4-B4^T^ were annotated using NCBI’s Prokaryotic Genome Annotation Pipeline (PGAP; Li W. et al., 2021). Metabolic pathways were predicted using the KEGG and RAST databases. The KEGG pathways were predicted using BlastKOALA server.^11^ The protein sequences from each genome were uploaded, and metabolic pathways were predicted for each isolate and reference strains using the prokaryotic database option. Subsequently, the BlastKOALA results were then processed using KEGG-decoder,^12^ employing the default parameters (Graham et al., 2018). From the KEGG pathway data, a heatmap was constructed using GraphPad Prism version 8.0.2. To compare the metabolic diversity among novel isolates and reference strains, we used the RAST server version 2.0^13^(Aziz et al., 2008). For the RAST annotation (see text footnote 13), we used the genome fasta nucleic acid file and applied the ‘RASTtk’ tool with the ‘automatically fix errors’ options on the RAST server. The annotated pathways of the three strains and reference strains from the RAST server were compared using two-tailed one-sample T-test method. Additionally, antiSMASH 6.01 was used to identify biosynthesis gene clusters (BGCs) and metabolic gene clusters (MGCs) within the genomes (Blin et al., 2019). The CRISPRCasFinder and CRISPRcasIdentifier^14^ servers were used to annotate the CRISPR Cas system (Couvin et al., 2018; Padilha et al., 2020). The presence of Cas enzymes were further confirmed by blasting each sequence in the UniProt database (The UniProt Consortium, 2023). The genomes of the three novel strains ABR2-5^T^, BKB1-1^T^, and WSW4-B4^T^ and three reference strains *R. agariperforans* DSM 26134^T^, *R. faecimaris* DSM 26133^T^, and *R. versicolor* DC003^T^ were analyzed to detect carbohydrate active enzymes (CAZymes) using the dbCAN2 meta server (Zhang et al., 2018). To detect polysaccharide utilization loci (PUL) in strains ABR2-5^T^ and BKB1-1^T^, we utilized the dbCAN-PUL database. For the identification of functional genes (*susC* and *susD*), the Prokka server was employed (Ausland et al., 2021). For the strain WSW4-B4^T^, we employed the dbCAN-PUL and PULDB databases^15^ to identify PUL and the presence of *susC* and *susD* genes. The PULDB database confirmed the existence of functional genes (*susC* and *susD*) in the proximity of CAZymes within PUL. Since the genomes of strains ABR2-5^T^ and BKB1-1^T^ were not available in the PULDB, we manually annotated them using the Prokka server.

### 2.6 Polysaccharide degradation testing

We tested the abilities of three strains to degrade complex polysaccharide by two methods. First, we cultivated three strains on solid media containing 60% (v/v) seawater, 0.01% (w/v) polypeptone, 1% (w/v) of each test polysaccharide, including cellulose, chitin, ĸ-carrageenan, λ-carrageenan, ι-carrageenan, inulin, laminarin, sodium alginate, starch, and xylan (Gao et al., 2017). For solidification, we used 0.6% (w/v) gellan gum instead of agar. For the degradation of agar and ĸ-carrageenan, agar and ĸ-carrageenan were used as solidifying agents instead of gellan gum. All three strains were inoculated on the media containing each test polysaccharide and then cultivated at 30°C for 7 days. The degradation of polysaccharides was detected by the production of a clear zone around the colonies or by the hydrolysis of solid media for the media containing agar and ĸ-carrageenan as solidifying agents. We used an iodine solution for the determination of starch hydrolysis. The hydrolysis of other polysaccharides was assessed by the development of a clear zone around the colonies.

Second, the degradation of the polysaccharide was further tested in liquid media with the same composition as the solid agar method, except that the media contained 0.2% (w/v) of each test polysaccharide (cellulose, chitin, ĸ-carrageenan, λ-carrageenan, ι-carrageenan, inulin, laminarin, sodium alginate, starch, and xylan). We inoculated two-day-old cultures of the three strains and cultivated them at 30°C in a shaking incubator. The cultures were harvested by centrifugation at 0, 3, and 7 days and then treated with 3,5-dinitrosalicylic acid (DNS) reagents, which reacted with the reducing sugars released from the degradation of polysaccharides. The change of color was measured by a microplate reader (Synergy H1, BioTek) at 570 nm after color development (Gao et al., 2017; Deshavath et al., 2020).

## 3 Results and discussion

### 3.1 Isolation and identification

**Figure 1:**
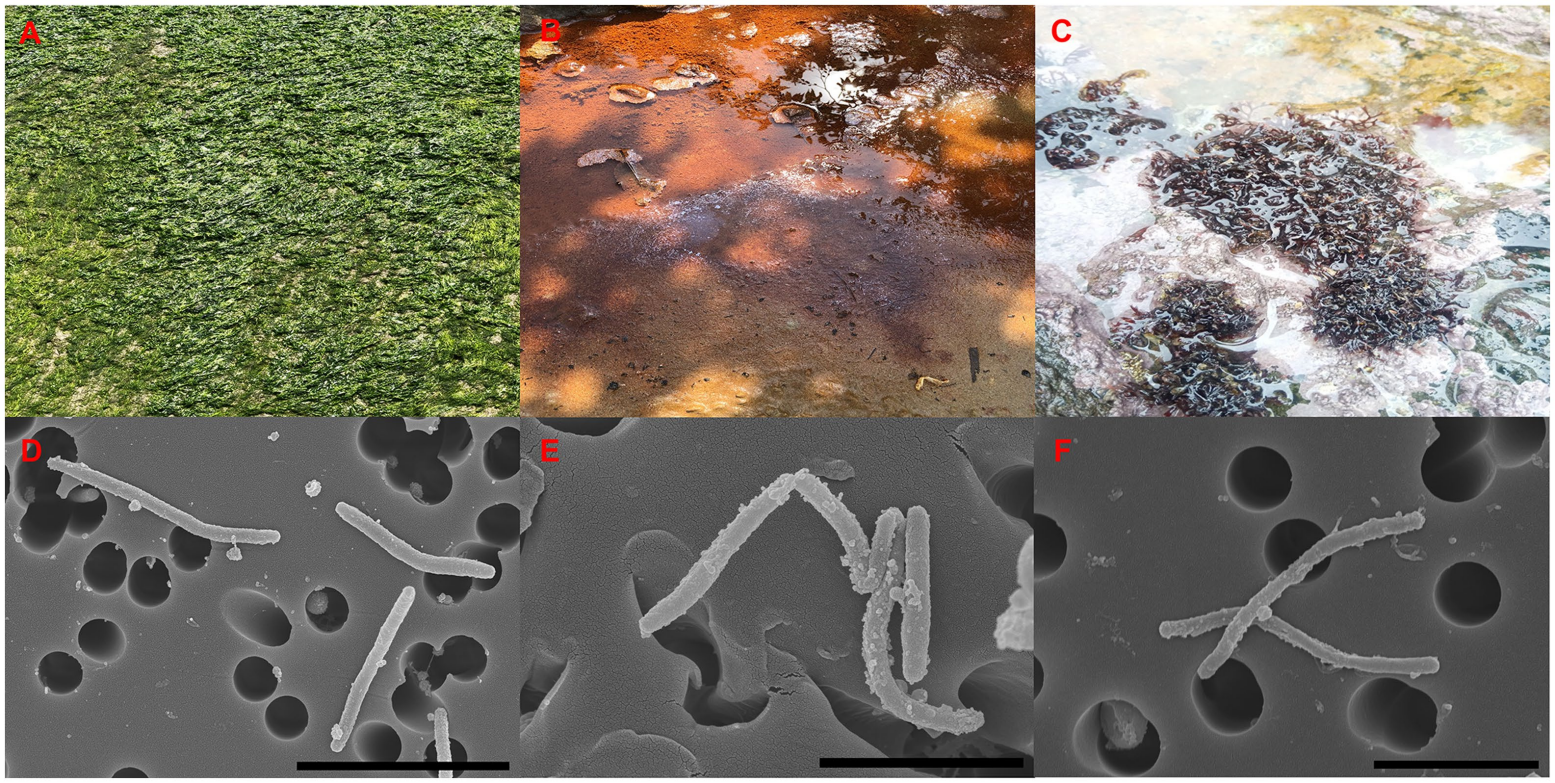
Sources of isolation **(A–C)**, and SEM images **(D–F)** of three isolates. Strain: ABR2-5^T^ **(A,D)**; BKB1-1^T^, **(B,E)**; WSW4-B4^T^ **(C,F)**. Scale bar: 4 μm **(D)**, 2 μm **(E,F)**.

The strains ABR2-5^T^, BKB1-1^T^, and WSW4-B4^T^ were isolated from a green algae of the *Ulva* sp., sea mud, and a red algae of the *Chondrus* sp., respectively (Figures 1A–C). All three strains grew well on MA and MVY. Colonies of strain ABR2-5^T^ and BKB1-1^T^ were orange, while the colonies of strain WSW4-B4^T^ were pale-yellow. All three strains produced round, smooth colonies with a diameter in a range of 9.8–16 mm. The size of the cells ranged from 2.1–3.8 μm in length and 0.22–0.31 μm in width (Figures 1D–F).

### 3.2 16S rRNA gene analysis

**Figure 2:**
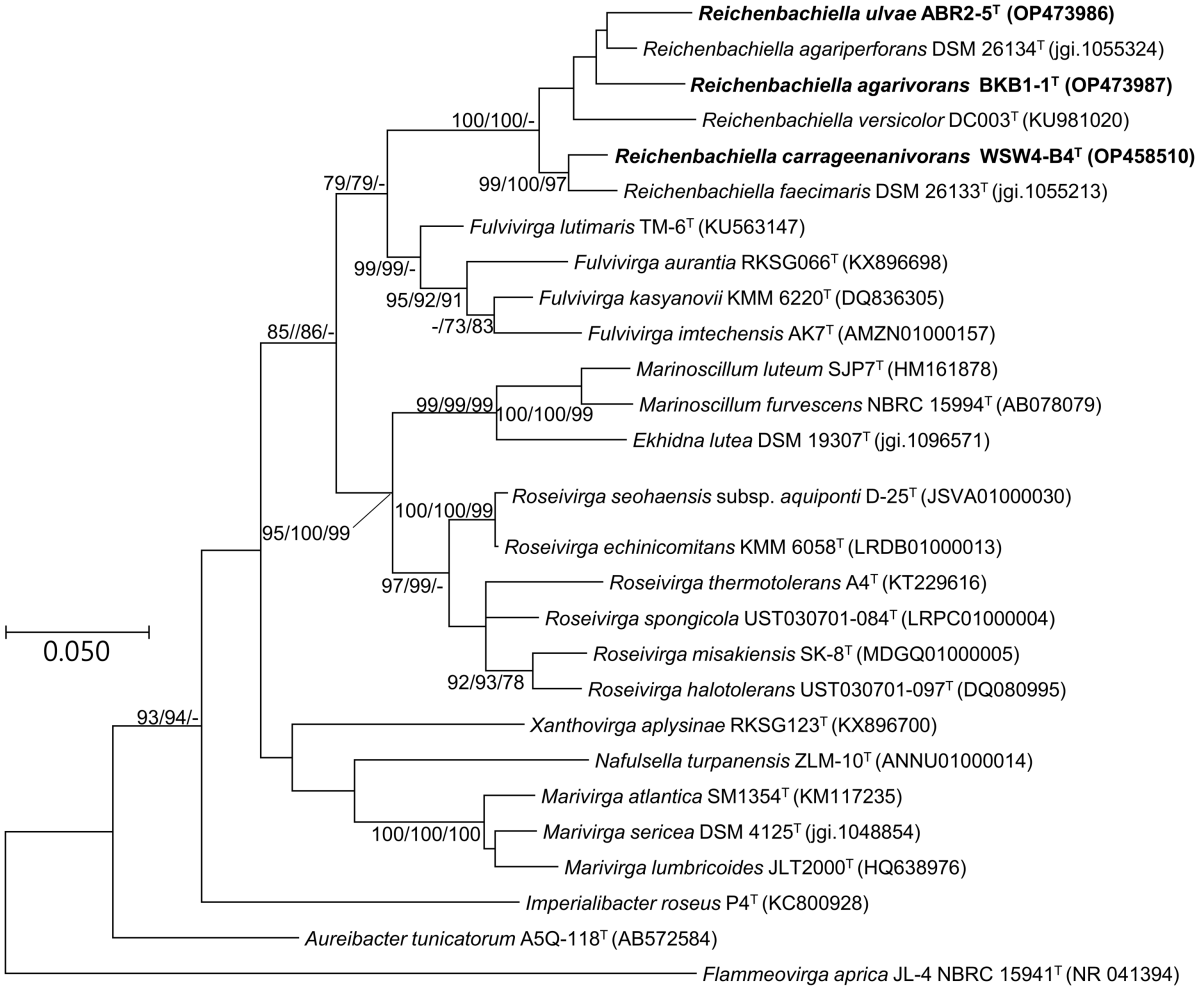
Maximum likelihood tree based on 16S rRNA gene sequences showing the phylogenetic relationship of three novel isolates and closely related genus belonging to the order Cytophagales. Bootstrap values (>70%) in the order of ML/NJ/MP are shown at the branch points based on 1,000 replications. GenBank accession numbers are shown in parentheses. Bar, 0.05 substitutions per nucleotide position. *Flammeovirga aprica* JL-4 NBRC 15941^T^ was used as an outgroup.

The phylogenetic position of the strains ABR2-5^T^, BKB1-1^T^, and WSW4-B4^T^ was determined based on the 16S rRNA gene sequences. All three strains were phylogenetically placed within the genus *Reichenbachiella*, which contains three species currently. The analysis of the 16S rRNA genes showed that the closest relatives of strains ABR2-5^T^ and BKB1-1^T^ were *R. agariperforans* DSM 26134^T^ with similarity values of 96.5% and 96.9%, respectively. Strain WSW4-B4^T^ was closest to *R. faecimaris* DSM 26133^T^ with a similarity value of 97.2%. In addition, the 16S rRNA gene similarity values among the three novel strains were less than 95%, while the values between the three novel strains and all three type strains in the genus *Reichenbachiella* were under 97% (Supplementary Table S1). These findings support the creation of three novel species. The threshold of the 16S rRNA gene similarity value is typically around 98.7% for species, 94.5% for genera, and 86.5% for families (Yarza et al., 2014). The phylogenetic tree based on 16S rRNA gene sequences (Figure 2) showed the clustering of three isolates (strains ABR2-5^T^, BKB1-1^T^, and WSW4-B4^T^) with three existing species of the genus *Reichenbachiella*: *R. agariperforans* DSM 26134 ^T^, *R. faecimaris* DSM 26133^T^, and *R. versicolor* DC003^T^. Interestingly, all the *Reichenbachiella* strains, including the three isolates formed a monophyletic clade with high bootstrap values (>70%). The strains *R. agariperforans* KCTC 12369^T^, *R. faecimaris* KCTC 82811^T^, and *R. versicolor* KCTC 82854^T^ were selected as the reference strains for the comparative taxonomic studies for three isolates. The 16S rRNA gene sequences of strains ABR2-5^T^, BKB1-1^T^, and WSW4-B4^T^ were registered in GenBank/EMBL/DDBJ with the accession numbers OP473986, OP473987, and OP458510, respectively.

### 3.3 Phenotypic analysis

All three isolates were Gram-staining-negative, long rod-shaped, and showed gliding motility. All three strains grew optimally at 15°C–30°C, but the strains ABR2-5^T^ and BKB1-1^T^ also grew at 37°C. Strain ABR2-5^T^ grew optimally at pH 6.0–9.0, while the optimum pH for the strains BKB1-1^T^ and WSW4-B4^T^ was 6.5–8.5. The NaCl concentration for the growth of all three isolates ranged from 0.5–4.0% (w/v), but only the strain ABR2-5^T^ showed growth up to 7% (w/v) NaCl and only the strain BKB1-1^T^ showed weak growth in the absence of NaCl. The strains ABR2-5^T^ and WSW4-B4^T^ could grow under only aerobic conditions, while strain BKB1-1^T^ could grow under both aerobic and anaerobic conditions. The detailed physiological characteristics of three novel strain and their reference strains are summarized in Table 1.

**Table 1:** Differential physiological characteristics of three novel isolates: ABR2-5^T^, BKB1-1^T^, and WSW4-B4^T^ and three existing type strains in the genus *Reichenbachiella: R. agariperforans* KCTC 12369^T^*, R. faecimaris* KCTC 82811^T^, and *R. versicolor* KCTC 82854^T^ Strains: 1, ABR2-5^T^; 2, BKB1-1^T^; 3, WSW4-B^T^; 4, *R. agariperforans* KCTC 12369^T^; 5, *R. faecimaris* KCTC 82811^T^; 6, *R. versicolor* KCTC 82854^T^.

Characteristics	1	2	3	4	5	6
Cell shape	Long rod	Rod	Rod	Rod	Rod	Rod
Colony morphology	Orange, smooth, circular, convex	Orange, colonies sunken into the agar	Pale yellow, colonies sunken into the agar	Orange, colonies sunken into the agar	Beige circular with regular edges	Circular, convex, translucent, dark brown
Width and length of cells (μm)	3.3–4.7	1.8–2.8	3.5–3.6	5.0–15.0	3.0–5.0	4.0–25.5
	0.3–0.4	0.2–0.3	0.2–0.3	0.5–0.7	0.3–0.5	0.2–0.4
Gliding motility	+	+	+	+	+	+
Flexirubin-type pigment	+	+	−	+	−	−
NaCl range (optimal) (%)	0.5–7	0.0–5	0.5–5	1.0–6.0	1.0–5.0	0.5–7.0
	(0.5–5.0)	(0.5–4.0)	(0.5–4.0)	(2.0)	(3.0)	(2.0–3.0)
Range of temperature (optimal) (°C)	10–37	10–37	(10–30)	4–35	5.0–35	10–37
	(15–37)	(15–30)	(15–30)	(25–28)	(28–30)	−28
Range of pH (optimal)	5.5–9.5	5.5–9.5	5.5–9.0	5.5–10.0	5.5–8.5	6.0–8.5
	(6.0–9.0)	(6.5–8.5)	(6.5–8.5)			(7.0–7.5)
Oxygen requirement	Aerobic	Facultative anaerobic	Aerobic	Aerobic	Aerobic	Aerobic

All the data are from this study except for the reference strains *R. agariperforans* DSM 26134^T^ (Nedashkovskaya et al., 2005), *R. faecimaris* DSM 26133^T^ (Cha et al., 2011), and *R. versicolor* DC003^T^ (Shi et al., 2018), which were obtained from the literature.

Cells of all three strains hydrolyzed Tween 20, not Tweens 40 and 80, and casein. The strains ABR2-5^T^ and BKB1-1^T^ were DNase-positive, while strain WSW4-B4^T^ was DNase-negative. Among the three strains only strains ABR2-5^T^ and BKB1-1^T^ contained flexirubin-type pigments. In API ZYM test, all three isolates showed activities of acid phosphatase, alkaline phosphatase, cystine arylamidase, α-chymotrypsin, esterase (C4), esterase lipase (C8), leucine arylamidase, naphthol-AS-BI-phosphohydrolase, trypsin, and valine arylamidase. All three strains were negative for α-fucosidase, α-glucosidase, and α-mannosidase. Among the three novel isolates only strain ABR2-5^T^ was positive for the activities of α-galactosidase and β-glucuronidase. The API 50CH tests were performed to differentiate the strains by determining their abilities to utilize sugars and produce acid molecules. A total of 50 sugar molecules were tested for hydrolysis, resulting in a color change due to acid production if the strains had the ability to metabolize it. The three novel strains possessed metabolic capabilities that distinguish them from the reference strains. All three strains produced acid from N-acetylglucosamine, L-fucose, D-galactose, and D-mannose, while the strain ABR2-5^T^ produced acid from additional amidon (starch), amygdalin, D-cellobiose, gentibiose, D-lactose, D-maltose, and L-rhamnose. Furthermore, in a GEN III MicroPlate (Biolog) test, all three novel strains utilized acetoacetic acid, D-cellobiose, L-fucose, gentiobiose, L-glutamic acid, D-mannose, and sodium butyrate. Although all three novel isolates and the three reference strains shared some biochemical characteristics, there are certain biochemical tests that were different between the three novel strains and also with the reference strains, as summarized in Supplementary Table S2.

The predominant fatty acids of the three novel strains and the three reference strains were iso-C_15:0_ in a range of 35.0–47.4%, C_16:1_ ω5c 8.9%–22.9%, and summed feature 3 (C_16:1_ ω7c/C_16:1_ ω6c) 9.0%–19.5%. Interestingly, among the three novel isolates only the strain ABR2-5^T^ had additional higher components of fatty acids C_16:0_ (13.0%), C_18:1_ ω9c (6.4%) and C_18:0_ (5.0%) as major fatty acids and the strain WSW4-B4^T^ had iso-C_15:1_\u00B0F (9.7%). The minor fatty acids of all three novel isolates and the reference strains were C_14:0_ (1.5%–2.7%), anteiso-C_15:0_ (1.0%–4.2%), C_16:0_ 3-OH (1.1%–3.0%), and iso-C_17:0_ 3-OH (1.7%–3.5%). Although the fatty acids among all three novel strains and the three reference strains were similar, the uniqueness and differentiation of fatty acids among all six strains are found and presented in Supplementary Table S3. The menaquinone 7 (MK-7) was detected as respiratory quinone which was also found in the reference strains in the genus *Reichenbachiella.* The quinone patterns are often conserved within genera or families of microorganisms. Thus, provide an important information for microbial classification and identification. Furthermore, quinones are essential cofactor in electron transport chains and participate in aerobic respiration.

The polar lipid profiles of the three novel isolates were similar to those of the type strains of the three-existing species of the genus *Reichenbachiella.* The three novel strains and the reference strains contained phosphatidylethanolamine (PE). In addition to PE, the strain ABR2-5^T^ had one aminophospholipid, one unidentified glycolipid, and seven unidentified lipids. The strain BKB1-1^T^ had two unidentified glycolipids and five unidentified lipids, while the strain WSW4-B4^T^ contained two aminophospholipids, two unidentified aminolipids, and five unidentified lipids. Among the three novel isolates, only the strain WSW4-B4^T^ had both aminophospholipid and aminolipid, the strain ABR2-5^T^ had only aminophospholipid, and strain BKB1-1^T^ had neither aminophospholipid nor aminolipid (Supplementary Figure S1).

### 3.4 Genomic general features and phylogeny

The complete genomes of the strains ABR2-5^T^, BKB1-1^T^, and WSW4-B4^T^ were determined using the Nanopore platform (Oxford Nanopore Technology, ONT). A CheckM analysis of three genomes of the novel isolates showed that the completeness of the three genomes ranged from 98.2–98.6%. The genome of the strain ABR2-5^T^ had 12 contigs with a total genome size 5.5 Mbp, among which contig 1 was the largest contig (5.4 Mbp), while the remaining 11 were short unassembled sequences. The strains BKB1-1^T^ and WSW4-B4^T^ each had one circular chromosome with sizes of 4.4 and 5.0 Mbp, respectively. The G + C contents of the three novel strains ranged from 41.8% to 42.0%, matching those of other species in the genus *Reichenbachiella*, which range from 37.1% to 43.4%. The genome was annotated with NCBI’s PGAP pipeline to annotate the total number of genes, CDS, rRNAs, and tRNAs, as summarized in Supplementary Table S4. The genomes of the strains ABR2-5^T^, BKB1-1^T^, and WSW4-B4^T^ are available in the NCBI with the GenBank accession numbers GCA_025833875, GCA_025502585, and GCA_025639805, respectively.

**Figure 3:**
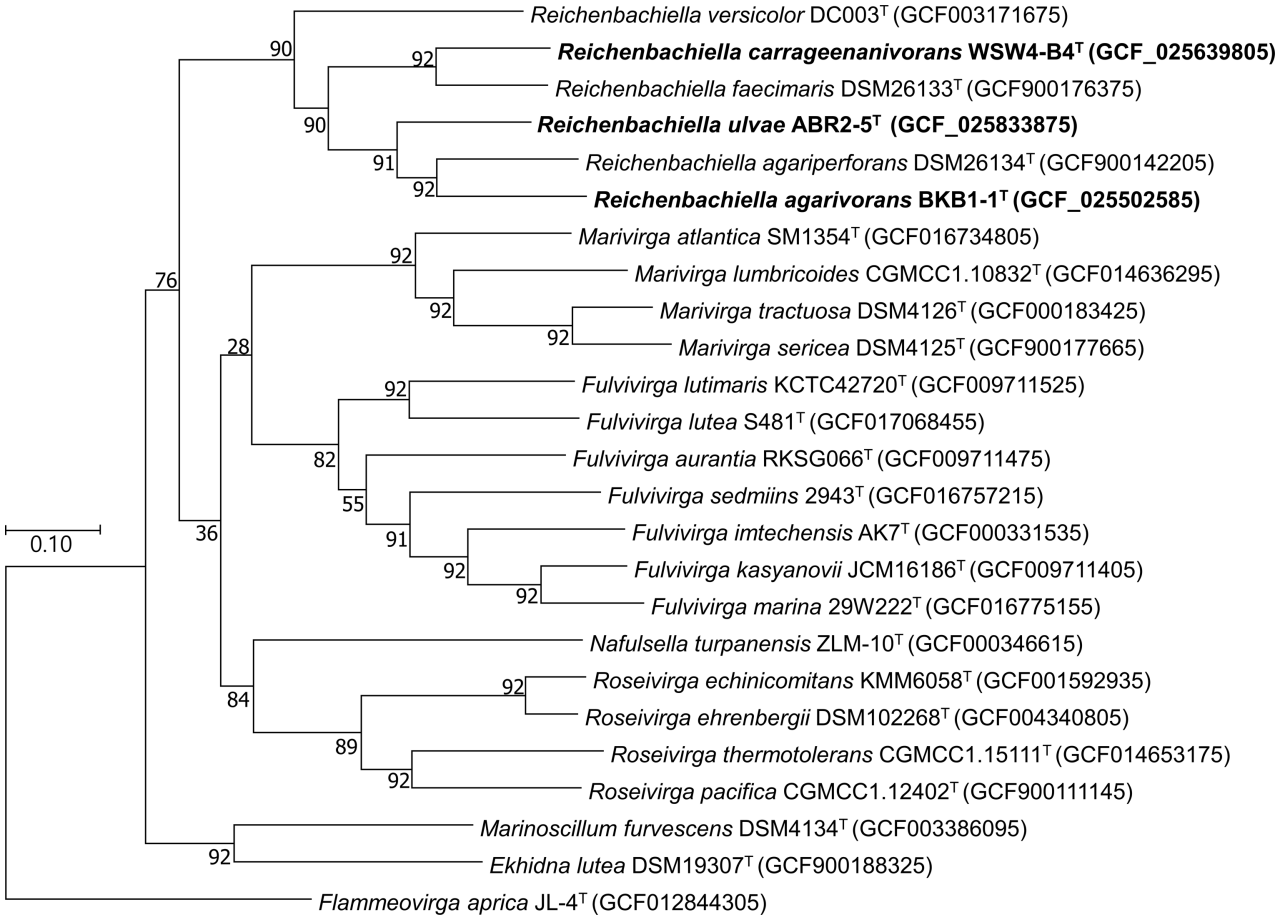
Maximum-likelihood phylogenetic tree exhibiting the relationships among the novel isolates ABR2-5^T^, BKB1-1^T^, WSW4-B4^T^ and closely related species, based on 92 core genes identified using the UBCG pipeline. GenBank accession numbers of the whole genome sequences are given in parentheses. *Flammeovirga aprica* JL-4^T^ (GCF012844305) was used as outgroups. Bootstrap values based on 1,000 replicates are indicated at the branch nodes. Bar, 0.1 substitutions per site.

To further confirm the taxonomic position of the three novel isolates, average nucleotide identity (ANI) and digital DNA–DNA hybridization (dDDH) were calculated among the isolates and the type strains of the existing species in the genus *Reichenbachiella*. The ANI and the dDDH values among the three novel strains and the three existing species were in ranges of 69.2%–75.5% and 17.7%–23.2% (Supplementary Table S5), respectively, which were significantly lower than the cut-off values of 95%–96% for the ANI value (Yoon et al., 2017b) and 70% for the dDDH value (Auch et al., 2010). Interestingly, the ANI and dDDH values among the three novel isolates were also lower than the cut-off values of ANI and dDDH for species differentiation, supporting the contention that all three novel isolates could be considered novel species. The genome-based phylogenetic tree also shows the robust clustering of the novel isolates with the three existing species: *R. agariperforans* DSM 26134^T^, *R. faecimaris* DSM 26133^T^, and *R. versicolor* DC003^T^ (Figure 3).

### 3.5 Genome functional analysis

**Figure 4:**
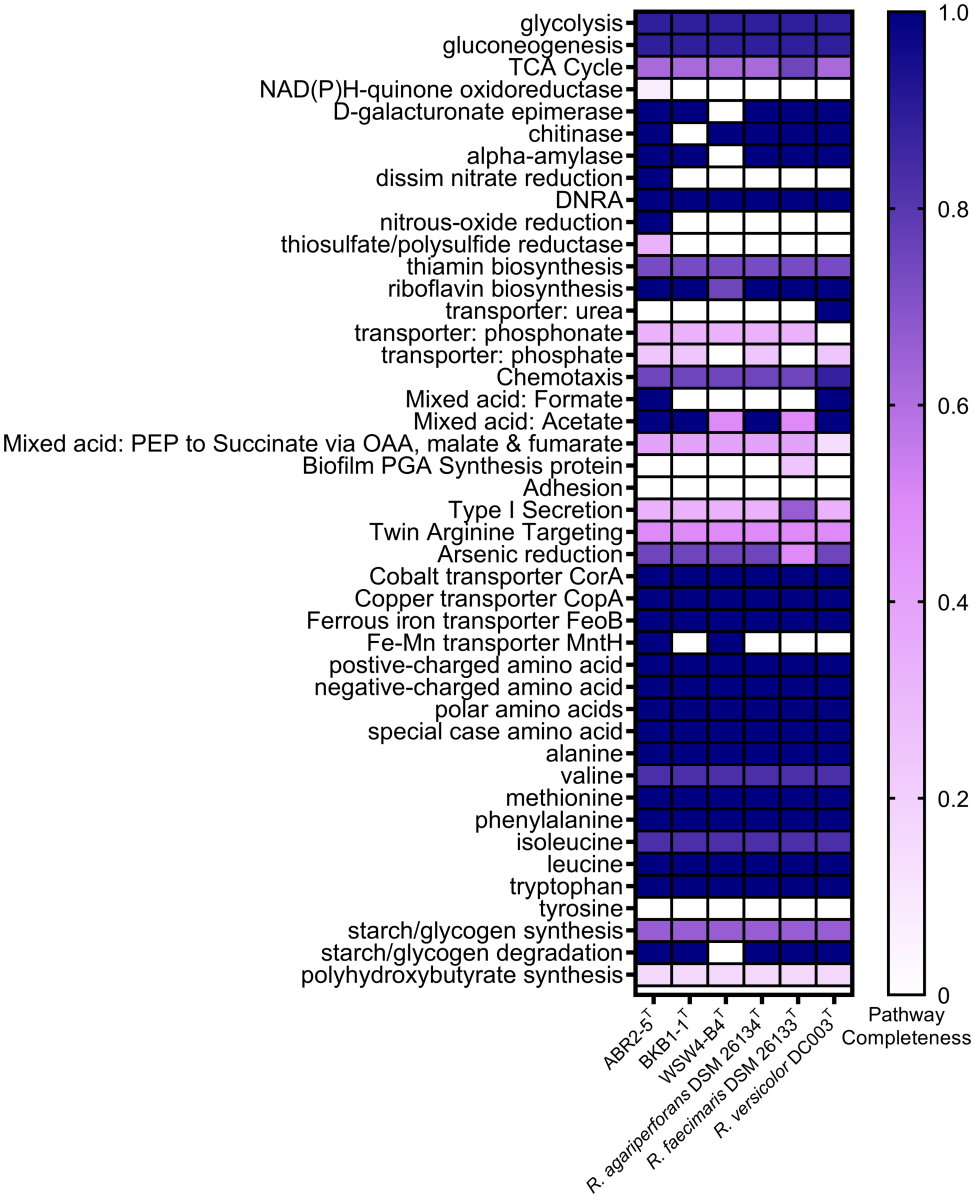
Heatmap of discriminated metabolic pathways within the genome of the three novel isolates and the reference strains in genus *Reichenbachiella.* The scale bar indicates that the intensity of color change reflects the completeness of pathways.

The whole genomes of the three novel strains and the three type strains of existing species were analyzed using various databases. First, the metabolic pathways were constructed using the BlastKOALA server, which utilizes the Kyoto Encyclopedia of Genes and Genomes (KEGG) pathway database. Then, only the enriched functions were plotted in the heatmap (Figure 4). The KEGG annotation showed that the strain ABR2-5^T^ had 71 pathways, which was the highest among the isolates. In contrast, the strains BKB1-1^T^ and WSW4-B4^T^ had 64 and 62 pathways, respectively. The presence of the highest number of metabolic pathways in the strain ABR2-5^T^ can be attributed to its larger genome size and the highest number of genes (Supplementary Table S4). Figure 4 shows that the central metabolic pathways, which commonly include aerobic respiration, sugar metabolism, and amino acid synthesis, displayed similarities among these strains. All six strains contained genes that participate in the synthesis of essential amino acids of leucine, methionine, phenylalanine, tryptophan, and valine, while interestingly none of the strains could synthesize tyrosine. The synthesis of essential amino acids in bacteria is crucial for their growth, survival, and adaptation to diverse environments. The KEGG analysis further highlighted that the strains also harbor genes for enzymes such as amylase, chitinase, and epimerase.

The genomes of the three strains ABR2-5^T^, BKB1-1^T^, and WSW4-B4^T^ carried three genes of heavy metal transporters: *CorA, CopA*, and *FeoB*, which is associated with cobalt, copper, and iron transportation (Zhang et al., 2009; Lau et al., 2016; Li et al., 2022). All three strains carried a part of pathway for reduction of arsenic. Moreover, the strains ABR2-5^T^ and WSW4-B4^T^ possessed additional *MntH* transporter genes, which play a role in the transportation of manganese and iron (Porcheron et al., 2013). The presence of heavy metal transporter genes in the genomes of three novel *Reichenbachiella* strains may confer advantages in terms of heavy metal tolerances, including arsenic, cobalt, copper, and manganese (Altimira et al., 2012).

The KEGG pathways further showed that the strains of the genus *Reichenbachiella* carry certain pathways that are important for the nitrogen cycle of coastal ecosystems. All the strains carry a complete pathway of dissimilatory nitrate reduction to ammonium (DNRA). The DNRA pathway is used by microbes to convert nitrate into ammonium, which is helpful to conserve bioavailable nitrogen within the ecosystem (Liu et al., 2023). DNRA is observed in various ecosystems, encompassing agricultural soils, marine sediments, and wastewater. DNRA not only conserves nitrogen within these ecosystems but also decreases the release of toxic greenhouse gases, such as nitrous oxide (Liu et al., 2021). Furthermore, the stimulation of DNRA has often been proposed as a strategy to improve fertilizer efficiency. In addition to DNRA pathways, the strain ABR2-5^T^ had additional pathways for reducing nitrate and nitrous oxide, which further highlights its role in the nitrogen cycle (Suenaga et al., 2018; Figure 4).

In the RAST system, the distribution of functional genes among the novel isolates and the type strains of existing species was similar. In all six strains, the largest number of genes were allocated to the metabolism of amino acids and its derivatives (211–253) and carbohydrates (122–168), followed by protein metabolism (109–170). Interestingly, the strain ABR2-5^T^ carries significantly higher numbers of genes for the metabolism of protein (170), carbohydrates (168), phosphorous (26), and potassium (13), whose *p* values were less than 0.05. The strains ABR2-5^T^ and WSW4-B4^T^ have four and one gene for iron acquisition and metabolism, respectively, while the strain BKB1-1^T^ lacks genes for iron acquisition and metabolism. Among the three novel isolates, the strain WSW4-B4^T^ has the most genes (15 genes) for regulation and cell signaling (Supplementary Table S6).

The antiSMASH analysis showed that the strains ABR2-5^T^, BKB1-1^T^, and WSW4-B4^T^ carry seven, six, and four biosynthetic gene clusters (BGCs) in their genomes, respectively (Table 2). All the novel isolates and reference strains carry type III polyketide synthases and terpenes except *R. faecimaris* DSM 26133^T^. The polyketide synthases constitute a group of multi-domain enzymes responsible for synthesizing polyketides, which are diverse group of secondary metabolites (Hochmuth and Piel, 2009), while terpenes are named for the number of five-carbon units that form their hydrocarbon skeleton that had significant biological activities (Helfrich et al., 2019). Among the three novel isolates, only the strains ABR2-5^T^ and BKB1-1^T^ have ectoine and arylpolyene type of BGCs, which may protect the bacterial strains under extreme conditions of salinity, drought, irradiation, pH, and temperature (Schöner et al., 2016; Bilstein et al., 2021).

**Table 2:** Biosynthetic gene clusters for secondary metabolites identified by antiSMASH of three isolates and three existing type strains in genus *Reichenbachiella.*

Strain	Metabolites	From (nt)	To (nt)	Most similar known cluster	Similarity (%)
1	Terpene	554,222	575,058	Carotenoid and Terpene	28
	Arylpolyene	2,734,426	2,780,398	Flexirubin and polyketide	22
	Phosphonate	2,808,331	2,841,707	Polysaccharide B and Saccharide	6
	Ectoine	3,977,552	3,987,941	Ectoine and Other	50
	Type III PKS	4,310,043	4,351,131	o and K-antigen, Saccharide	4
	Type I PKS	5,338,720	5,385,913		
	Resorcinol	5,934,952	5,481,028	Flexirubin and polyketide	13
2	Arylpolyene & Resorcinol	206,645	251,834	Flexirubin and Polyketide	16
	RRE-containing domain	375,672	396,118		
	Ectoine	1,240,170	1,250,568	Ectoine and Other	50
	Type III PKS	1,582,227	1,623,315		
	Type I PKS	2,141,220	2,188,521		
	Arylpolyene	2,474,320	2,520,790	Flexirubin and Polyketide	25
	Terpene	4,207,889	4,228,725	Carotenoid and Terpene	28
3	Type III PKS	903,303	944,394		
	RRE-containing domain	1,686,875	1,707,312		
	Terpene	3,200,818	3,221,654	Carotenoid and Terpene	28
	Phosphonate	3,757,063	3,771,651	Polysaccharide B and Saccharide	6
4	Arylpolyene & Resorcinol	1	32,142	Flexirubin and Polyketide	16
	Terpene	619,422	640,258	Carotenoid and Terpene	28
	Ectoine	684,849	695,250	Ectoine and Other	50
	Arylpolyene	95,289	141,558	Flexirubin and Other	44
	Type III PKS	242,032	283,120		
	Type I PKS	94,015	141,178		
5	Phosphonate	53,093	67,561	Polysaccharide B and Saccharide	6
	Type III PKS	1,011,891	1,052,988		
	Arylpolyene	589,135	625,381	Flexirubin and Polyketide	22
6	Other	839,401	880,930		
	Terpene	924,027	944,866	Carotenoid and Terpene	28
	NRPS-independent-siderophore	16,275	31,047	Bisucaberin	83
	NRPS-like	414,939	459,927		
	Terpene	224,736	245,515		

Nucleotide positions with each genome are presented. Strains: 1, ABR2-5^T^; 2, BKB1-1^T^; 3, WSW4-B4^T^; 4, *R. agariperforans* DSM 26134^T^; 5, *R. faecimaris* DSM 26133^T^; 6, *R. versicolor* DC003^T^ NRPS, Nonribosomal Peptide Synthetase; PKS, Polyketide Synthases; RRE, RiPP (Ribosomally Synthesized and Post-Translationally Modified Peptides) Recognition Element.

^*^From, indicates the gene’s initiation position of biosynthetic gene cluster (BGC). ^**^To, indicates the gene’s termination positions of biosynthetic gene cluster (BGC).

### 3.6 CRISPR-Cas analysis

The CRISPR-Cas system consists of a CRISPR array and Cas cascade. The Cas cascade is a circuit of CRISPR-associated (Cas) enzymes (Brouns et al., 2008). The Cas cascade of all three isolated strains contains Cas9, Cas1, and Cas2 in order of upstream to downstream. According to the current guideline of CRISPR-Cas classification, all the isolated strains contain the class 2 type II subtype C1 CRISPR-Cas system (Makarova et al., 2020). The Cas1 and Cas2 of all three isolated strains were similar. According to the UniProt database it shows highest similarity to *Reichenbachiella* sp. 5 M10, which was isolated from Japan’s West Sea. It can be inferred that Cas1 and Cas2 are conserved in the genus *Reichenbachiella*.

Meanwhile, the Cas9 of each isolate was varied. According to the UniProt database the highest similarity of ABR2-5^T^ was with *Aquaticitalea lipolytica* (the class *Flavobacteriia*; Supplementary Table S7); BKB1-1^T^ showed highest similarity with *Acidiluteibacter ferrifornacis* (the class *Flavobacteriia*; Supplementary Table S8); and WSW4-B4^T^ showed highest similarity with *Reichenbachiella* sp. 5 M10 (the class *Cytophagia*; Supplementary Table S9).

### 3.7 Polysaccharide degradation

#### 3.7.1 Prediction of CAZyme gene clusters and carbohydrate-active enzymes using dbCAN meta server

**Figure 5:**
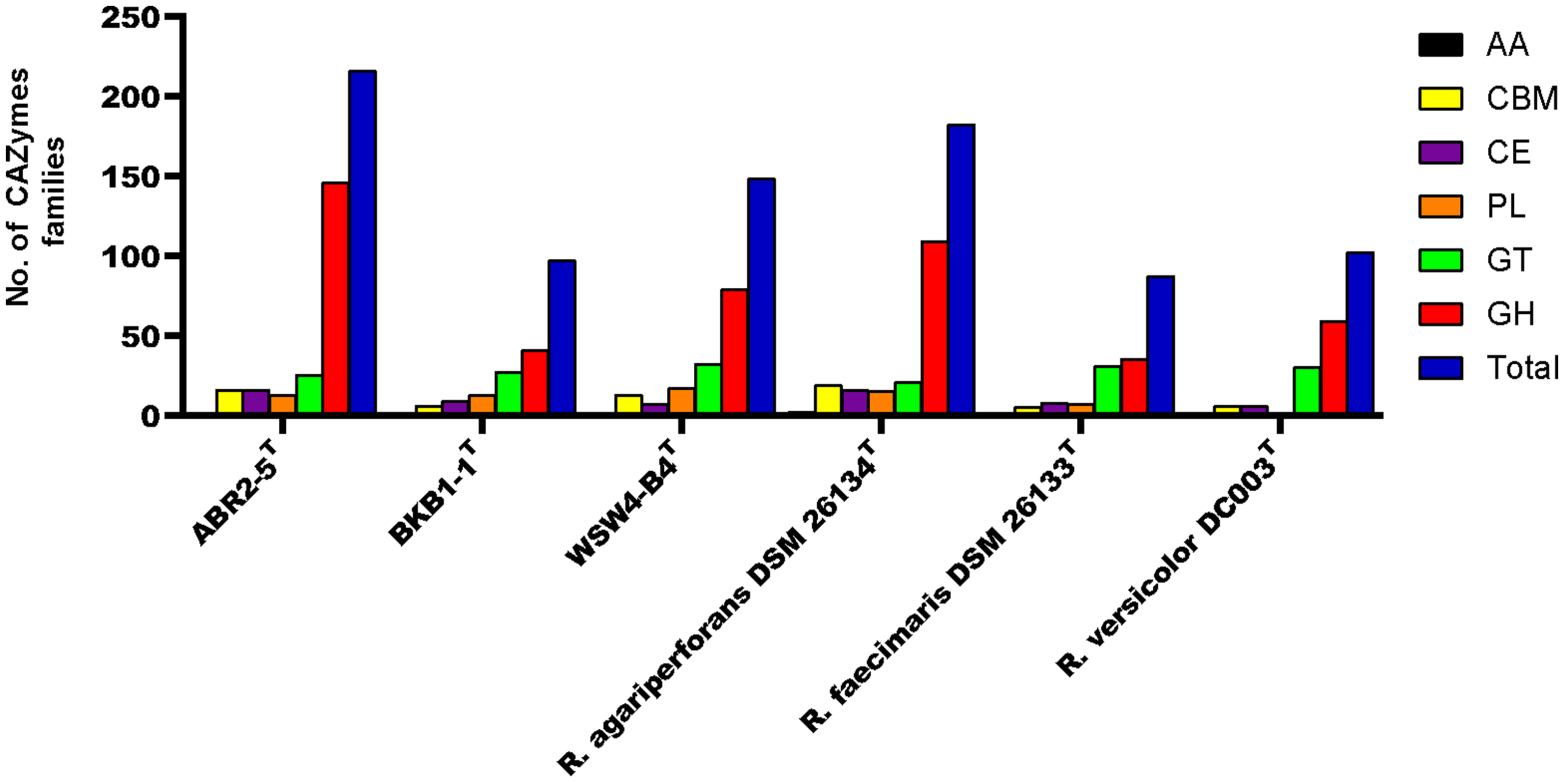
The carbohydrate-active enzyme (from dbCAN2 meta server) compositions of three novel isolates and reference strains in genus *Reichenbachiella.*

CAZyme gene cluster (CGC) is a genomic region that consists of carbohydrate-active enzymes (CAZymes), transporter, signal transduction, and transcription factor genes (Ameri et al., 2022). The CGCs within the genomes of the strains were identified using the CGC finder tool in the dbCAN meta server. The CGC finder tool detected a total of 67 CGCs in the genome of the strain ABR2-5^T^, while 49 and 66 CGCs were detected in the genomes of the strains BKB1-1^T^ and WSW4-B4^T^, respectively. Next to CGCs detection, the number of CAZymes was predicted in all three isolates and reference strains of the genus *Reichenbachiella*. The genome of the strain ABR2-5^T^ contains 216 CAZymes, which is a higher number than other strains in the genus *Reichenbachiella*. The 216 CAZymes consist of 146 glycoside hydrolases (GHs), 13 polysaccharide lyases (PLs), 16 carbohydrate esterases (CEs), 25 glycosyltransferases (GTs), and 16 carbohydrate-binding modules (CBM). The genomes of the strains BKB1-1^T^ and WSW4-B4^T^ contain 97 and 148 CAZymes that are distributed to 41 GHs, 13 Pls, 9 CEs, 27 GTs, and 3 CBMs in the strain BKB1-1^T^ and 109 GHs, 15 PLs, 7 CEs, 21 GTs, and 13 CBMs in the strain WSW4-B4^T^. The distribution of CAZymes in the novel and the reference type strains is presented in Figure 5. The percentage of CAZyme genes out of the total genes and the ratio of glycoside hydrolases (GHs) per Mbp genome were calculated. Among the three isolates, the strain ABR2-5^T^ carries a higher amount (4.68%) of CAZymes, while the strains BKB1-1^T^ and WSW4-B4^T^ carry 2.61% and 3.84% CAZymes in their genomes, respectively. The strain ABR2-5^T^ carries 26.55 GHs per Mbp in the genome, while the strains BKB1-1^T^ and WSW4-B4^T^ carry 9.32 and 15.80 GHs per Mbp in the genome (Supplementary Table S10). The number of CAZymes in the stain ABR2-5^T^ was significantly higher than that of the other strains in the phylum *Bacteroidota*, such as *Formosa agariphila* KMM 3901^T^, totaling 193 CAZymes (Mann et al., 2013), and *Cellulophaga algicola* IC166^T^, totaling 101 CAZymes (Abt et al., 2011), while being closest to *Zobellia* sp. (257–315 CAZymes), which is a known polysaccharide degrader (Thomas et al., 2017; Chernysheva et al., 2019).

#### 3.7.2 Prediction of polysaccharide utilization loci using dbCAN-PUL server and PULDB

PULs refer to the cluster of genes consisting of CAZymes and other genes that participate in the digestion and utilization of complex polysaccharides. The prediction of PUL regions in the genome was confirmed by a BLASTX search against the dbCAN-PUL database, which contains experimentally verified PULs from 173 bacterial species of 10 different bacterial phyla (Ausland et al., 2021). By using a BLASTX search, the genomes of isolates for those with PULs % sequence identity values ranging from 18 to 91% were detected. Among the predicted PULs, only those PULs that had % sequence identity values greater than 74% were selected for further analysis (Wu et al., 2021). The analysis showed that strains WSW4-B4^T^ and BKB1-1^T^ have nine and seven PULs, respectively, whose similarity values were above 74%, while the remaining strains in the genus *Reichenbachiella* have PULs whose value of identity was less than 74%.

Interestingly, both the strains BKB1-1^T^ and WSW4-B4^T^ could degrade agar and alginate (see below section 3.8.3) and harbor 3–4 PULs (PUL0316, PUL0460) for the degradation of agar and 1–3 PULs (PUL0151, PUL0235, PUL0313) for the degradation of alginate, respectively.^16^ The strain WSW4-B4^T^ additionally degraded ĸ-carrageenan and carries PUL (PUL0148), which may be involved in the degradation of ĸ-carrageenan (Gobet et al., 2018). All three novel isolates could degrade laminarin, but only the strains BKB1-1^T^ and WSW4-B4^T^ have PULs (PUL0005, PUL0314) that may participate in the degradation of laminarin (Thomas et al., 2017; Table 3).

**Table 3:** Number of polysaccharide-degrading genes based on dbCAN meta server and dbCAN-PUL, and *in vitro* activities of three novel strains of genus *Reichenbachiella* Strains: 1, ABR2-5^T^; 2, BKB1-1^T^; 3, WSW4-B4^T^.

Strain	Polysaccharides	CAZyme families^*^	PUL^**^	*In vitro* degradation
1	Agarose	GH50 (0)		−
	Alginate	PL6 (0), PL7 (0)		−
	Cellulose	GH5 (9), GH9 (1)		−
	Chitin	GH18 (2) GH20 (3)		−
	k-Carrageenan	GH16 (2)		−
	λ-Carrageenan	GH110 (5)		−
	ι-Carrageenans	GH82 (0)		−
	Laminarin	GH16 (2),		+
	Starch	GH13 (3) GH57 (0)		+
	Xylan	GH3 (16)		−
2	Agarose	GH50 (2), GH86 (1)	PUL0316 (2), PUL0460 (1)	+
	Alginate	PL6 (1), PL7 (3),	PUL0151 (1)	+
	Cellulose	GH5 (0), GH9 (0)		−
	Chitin	GH20 (1)		−
	k-Carrageenan	GH16 (4)		−
	λ-Carrageenan	GH110 (0)		−
	ι-Carrageenans	GH82 (0)		−
	Laminarin	GH16 (4),	PUL0314 (1)	+
	Starch	GH13 (1) GH57 (0)		+
	Xylan	GH3 (3)		+
3	Agarose	GH50 (2), GH86 (2)	PUL0316 (3), PUL0460 (1)	+
	Alginate	PL6 (3), PL7 (3),	PUL0151 (1), PUL0235(1), PUL0313 (1)	+
	Cellulose	GH5 (0), GH9 (0)		−
	Chitin	GH18 (2) GH20 (2)		−
	k-Carrageenan	GH16 (6)	PUL0148 (1)	+
	λ-Carrageenan	GH110 (5)		+
	ι-Carrageenans	GH82 (0)		−
	Laminarin	GH16 (6),	PUL0005 (1)	+
	Starch	GH13 (0) GH57 (1)		−
	Xylan	GH3 (5)		−

^*^CAZyme families with the number of genes in parentheses that are expected to participate in degradation of polysaccharides. ^**^PULs (polysaccharide utilization loci) with the number of PULs in parentheses that are expected to participate in degradation of polysaccharides.

PULs were also identified by the presence of iconic functional genes such as *susC* and *susD* in the vicinity of CAZymes. For the strains ABR2-5^T^ and BKB1-1^T^, we used the Prokka server to search PULs. The analysis revealed the presence of *susC* and *susD* upstream of one GH13 in the genome of strain ABR2-5^T^. However, *susC* and *susD* were not detected in the proximity of the other GH16. It is possible that laminarin degradation occurred through the action of GH16, utilizing *susC* and *susD* from other PULs via cross-utilization of transport systems. In case of strain BKB1-2^T^, multiple instances of *susC* and *susD* were detected within its genome. However, these transport genes were not organized in a circuitry, indicating that the transport gene were not found next to each other in the same direction.

To detect PULs based on functional genes (*susC* and *susD*) in the strain WSW4-B4^T^, we used the PULDB database. The analysis revealed the presence of *sucC* and *susD* in the vicinity of CAZymes. Specifically, *sucC* and *susD* were detected in the proximity of GH50, GH16, GH110, and PL6, enabling the strain to degrade agarose, laminarin, κ-carrageenan, λ-carrageenan, and alginate, respectively.

#### 3.7.3 *In vitro* polysaccharide degradation

The degradation of polysaccharides of agar, cellulose, chitin, ĸ-carrageenan, λ-carrageenan, ι-carrageenan, laminarin, sodium alginate, starch, and xylan was tested both in solid and liquid media. First, degradation of the test polysaccharides was evaluated on solid media by detecting a clear zone around the colonies and by liquefaction of the solid media. Among the three novel isolates, the strain WSW4-B4^T^ could degrade agar, sodium alginate, laminarin, ĸ-carrageenan and, λ-carrageenan; the strain BKB1-1^T^ could degrade agar, starch, sodium alginate, and laminarin while the strain ABR2-5^T^ degraded only laminarin and starch (Table 3). The enzyme assay for the degradation of cellulose, chitin, ĸ-carrageenan, λ-carrageenan, ι-carrageenan, laminarin, sodium alginate, starch, and xylan were performed by detecting reducing sugars in the culture broth by a 3, 5-dinitrosalicylic acid assay. The assay was conducted over three time points of cultivation: at the start (day 0), after 3 days, and after 7 days. The changes in optical density were measured using a microplate reader. The increase of optical density indicated the presence of reducing sugars. The results showed that the strain WSW4-B4^T^ degraded sodium alginate, laminarin, ĸ-carrageenan, and λ-carrageenan. The strain BKB1-1^T^ produced reducing sugars from the degradation of sodium alginate, laminarin, and starch, while the strain ABR2-5^T^ produced reducing sugars only from the degradation of laminarin and starch.

The *in vitro* degradation of each test polysaccharide was supported by the *in silico* detection of CAZymes and PULs in the genomes of all three novel isolates. First, the degradation of starch and laminarin was supported by the presence of a high abundance of GH13, which is primarily responsible for α-amylase (Janeček et al., 2014) and GH57 and GH13 for laminarin (Cantarel et al., 2009). The strains BKB1-1^T^ and WSW4-B4^T^ could degrade sodium alginate. Interestingly, in the genome of the strain BKB1-1^T^ there were one PL6 and three PL7, while WSW4-B4^T^ had three PL6 and three PL7, which may have role in the breakdown of alginate (Li Q. et al., 2021). Furthermore, strains BKB1-1^T^ and WSW4-B4^T^ carry one GH16, GH50, and GH86, which may participate in the degradation of agar (Gao et al., 2017). Among all the novel isolates, strain WSW4-B4^T^ was able to degrade ĸ-carrageenan and λ-carrageenan. Interestingly, in the genome of strain WSW4-B4 ^T^, we found six GH16 and five GH110 genes, which indicate the strain’s capability for the degradation of ĸ-carrageenan and λ-carrageenan, respectively (Gao et al., 2017; McGuire et al., 2020). The details of each test polysaccharide, the CAZyme families, the numbers of PULs, and *in vitro* test activities are summarized in Table 3.

The three novel strains possessed the ability to degrade various complex polysaccharides such as agar, alginate, carrageenan, laminarin, and starch. Genome analysis further highlighted that all three novel strains carry a high number of CAZymes for the degradation of complex polysaccharides. The degradation of these complex polysaccharides can produce oligosaccharides with practical applications in biomedicine, cosmetics and food industry (Jutur et al., 2016). For example, alginate and laminarin oligosaccharides exhibit various biological activities, including antioxidant, antitumor, and immunomodulatory effects (Zargarzadeh et al., 2020). Our study isolated and characterized three new *Reichenbachiella* strains which harbor various carbohydrate active enzymes that can be utilized for the production of biologically active oligosaccharides.

In conclusion, during a study on the microbial diversity of the West Sea, Korea, three novel stains were isolated from algae and sea mud samples. Through a polyphasic approach, the three strains were determined to be affiliated with the genus *Reichenbachiella* of the phylum *Bacteroidota*. The presence of a high number of CAZymes in the genomes of these strains, which enable the degradation of complex polysaccharides, suggests that they have the potential to serve as effective polysaccharide degraders. Furthermore, the strains carry certain genes involved in the synthesis of essential amino acids and vitamins, secondary metabolites, and also carry important pathways for heavy metal metabolism and for the nitrogen cycle.

### 3.8 Description of *Reichenbachiella ulva*e sp. nov.

*Reichenbachiella ulvae* (ul’vae. L. gen. n. *ulvae*, of the seaweed genus *Ulva*)

Cells are Gram-strain-negative, rod-shaped, strictly aerobic, and oxidase-and catalase-positive. The colonies of MA are circular, smooth, and orange in color. The strain grows at temperature 10–37°C (optimum, 30°C), at pH 5.5–9.5 (optimum, pH 7.0), and with 0.5–7% NaCl (optimum, 2%). Positive for the degradation of laminarin and starch. Positive for fexirubin-type pigment. The predominant fatty acid components are iso-C_15:0_, C_16:1_ ω5c, and C_16:0_.

The type strain, ABR2-5^T^ (=KCTC 82990^T^ = JCM 35839^T^), was isolated from the green alga *Ulva* sp. For the type stain the G + C content is 42.0%.

### 3.9 Description of *Reichenbachiella agarivorans* sp. nov.

*Reichenbachiella agarivorans* (a.ga.ri.vo’rans. N.L. neut. n. *agarum*, agar; L. pres. part. *vorans*, devouring; N.L. part. Adj. *agarivorans*, agar-devouring)

Cells are Gram-strain-negative, rod-shaped, facultative anaerobe, and oxidase and catalase positive. The colonies are orange in color and sunken into on MA. The strain grows at temperature 10–37°C (optimum, 30°C), at pH 5.5–9.5 (optimum, pH 7.0), and with 0.5–5% NaCl (optimum, 2%). Positive for the degradation of agar, alginate, laminarin, and starch. Positive for fexirubin-type pigment. The predominant fatty acid components are iso-C_15:0_, C_16:1_ ω5c, and summed feature 3 (C_16:1_ ω7c/C_16:1_ ω6c).

The type strain, BKB1-1^T^ (=KCTC 82964^T^ = JCM 35840^T^), was isolated from sea mud. For the type stain the G + C content is 42.1%.

### 3.10 Description of *Reichenbachiella carrageenanivorans* sp. nov.

*Reichenbachiella carrageenanivorans* (car.ra.gee.na.ni.vo’rans. N.L. neut. n. *carrageenanum*, carrageenan; L. v. *voro*, to devour; N.L. part. Adj. *carrageenanivorans*, carrageenan-devouring)

Cells are Gram-strain-negative, rod-shaped, strictly aerobe, and oxidase and catalase positive. The colonies are pale-yellow and sunken into the agar. The strain grows at temperature 10°C–30°C (optimum, 30°C), at pH 5.5–9.0 (optimum, pH 7.0), and with 0.5%–5% NaCl (optimum, 2%). Positive for the degradation of agar, alginate, ĸ-carrageenan, λ-carrageenan, and laminarin. Negative for fexirubin-type pigments production. The predominant fatty acid components are iso-C_15:0_, summed feature 3 (C_16:1_ ω7c/C_16:1_ ω6c), C_16:1_ ω5c, and iso-C_16:0_\u00B0F. The type strain, WSW4-B4^T^ (=KCTC 82706^T^ = JCM 35841^T^), was isolated from the red algae *Chondrus* sp. For the type stain the G + C content is 41.8%.

## Data availability statement

The original contributions presented in the study are included in the article/Supplementary material, further inquiries can be directed to the corresponding author.

## Author contributions

NM: Conceptualization, Investigation, Methodology, Writing – original draft, Writing – review & editing. FA: Formal Analysis, Investigation, Visualization, Writing – original draft. ON: Formal Analysis, Validation, Writing – review & editing. S-GK: Conceptualization, Funding acquisition, Methodology, Project administration, Supervision, Writing – review & editing.

## Footnotes

^1^ https://lpsn.dsmz.de/phylum/bacteroidota

^2^ https://lpsn.dsmz.de/order/cytophagales

^3^ https://lpsn.dsmz.de/family/reichenbachiellaceae

^4^ https://lpsn.dsmz.de/genus/reichenbachiella

^5^ http://www.ezbiocloud.net

^6^ github.com/fenderglass/Flye

^7^ github.com/Ecogenomics/CheckM

^8^busco.ezlab.org/

^9^ www.ezbiocloud.net/tools/ani

^10^ https://ggdc.dsmz.de/ggdc.php

^11^ kegg.jp/blastkoala/

^12^ github.com/bjtully/BioData/tree/master/KEGGDecoder

^13^rast.nmpdr.org/

^14^ https://github.com/BackofenLab/CRISPRcasIdentifier

^15^ http://www.cazy.org/PULDB/

^16^ https://bcb.unl.edu/dbCAN_PUL/Repository
