# Supplementary material for: Three novel marine species of the genus Reichenbachiella exhibiting degradation of complex polysaccharides
Source: Front Microbiol. 2023 Dec 14;14:1265676. doi: 10.3389/fmicb.2023.1265676 (PMC10752948; doi:10.3389/fmicb.2023.1265676)
Supplement: Supplementary file 1 [file Data_Sheet_1.PDF]

Three novel marine species of the genus *Reichenbachiella* exhibiting degradation of complex polysaccharides

Neak Muhammad[1, 2], Forbes Avila[1, 2], Olga I. Nedashkovskaya[3], Song-Gun Kim[1, 2]*

[1]Biological Resource Center/Korean Collection for Type Cultures (KCTC), Korea Research Institute of Bioscience and Biotechnology, 181 Ipsingil, Jeongeup, Jeonbuk 56212, the Republic of Korea

[2]Department of Environmental Biotechnology, KRIBB School of Biotechnology, University of Science and Technology (UST), 217 Gajeong-ro, Yuseong, Daejeon 34113, the Republic of Korea

[3]G.B. Elyakov Pacific Institute of Bioorganic Chemistry of the Far-Eastern Branch of the Russian Academy of Sciences, Prospekt 100 Let Vladivostoku 159, 690022 Vladivostok, Russia

***Corresponding author:**

Song-Gun Kim

Email: sgkim@kribb.re.kr

Figure S1. Two-dimensional thin-layer chromatography showing the polar lipids profile of three isolates

A, strain ABR2-5^{T}; **B,** strain BKB1-1^{T}; **C,** strain WSW4-B^{T}

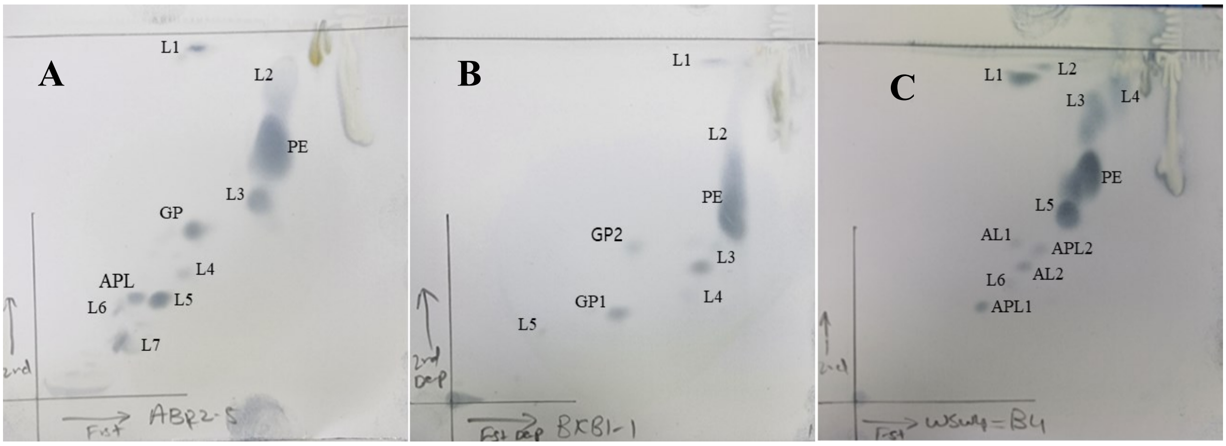

The plate was sprayed by molybdatophosphoric acid to detect total lipids

AL, unidentified aminolipid; APL, unidentified aminophospholipid; PE, phosphatidylethanolamine; PL, unidentified phospholipid; GL, unidentified glycolipid; L, unidentified lipid.

Table S1. Similarity values of the 16S rRNA gene among three isolates and all three existing species in genus *Reichenbachiella*.

Strains: 1, ABR2-5^T; 2, BKB1-1^T; 3, WSW4-B4^T; 4, *R. agariperforans* DSM 26134^T; 5, *R. faecimaris* DSM 26133^T; *R. versicolor* DC003^T

	**1**	**2**	**3**	**4**	**5**	**6**
**1**	1.000	0.947	0.944	0.965	0.949	0.940
**2**	0.947	1.000	0.940	0.961	0.939	0.936
**3**	0.944	0.940	1.000	0.948	0.971	0.936
**4**	0.965	0.961	0.948	1.000	0.945	0.946
**5**	0.949	0.939	0.971	0.945	1.000	0.942
**6**	0.940	0.936	0.936	0.946	0.942	1.000

Table S2. Differential biochemical characteristics of three novel strains and reference strains in the genus *Reichenbachiella*

Strains: 1, ABR2-5^T; 2, BKB1-1^T; 3, WSW4-B^T; 4, *R. agariperforans* KCTC 12369^T; 5, *R. faecimaris* KCTC 82811^T; 6, *R. versicolor* KCTC 82854^T

All data are from this study. All strains were oxidase and catalase positive and positive for the hydrolysis of Tween 20.

+, Positive; -, negative; w, weak

**Biochemical tests**	**1**	**2**	**3**	**4**	**5**	**6**
DNase activity	+	+	-	+	-	-
Hydrolysis of Tween 80	-	-	-	-	+	-
**Enzyme activity (API ZYM)**						
Trypsin	+	+	+	w	w	+
β-Galactosidase	+	+	-	+	-	-
β-Glucuronidase	-	+	-	-	-	-
β-Glucosidase	+	w	-	-	-	-
N-Acetyl-*β*-glucosaminidase	+	w	+	+	+	-
**API 50 CH acid production**						
D-Arabinose	-	-	-	+	-	-
L-Arabinose	-	-	-	+	-	-
N-Acetyl-glucosamine	+	+	+	+	-	-
Amygdalin	+	+	-	+	-	-
D-Adonitol	-	-	+	-	-	-
Amidon (starch)	+	-	-	+	-	-
D- Cellobiose	+	+	-	+	-	-
L-Fucose	+	+	+	+	-	-
Glycogen	+	-	-	+	-	-
Gentiobiose	+	+	-	+	-	-
D-Galactose	+	+	w	+	+	-
D-Lactose	+	-	-	+	-	-
D-Mannose	+	+	+	+	+	-
D-Maltose	+	-	-	+	-	-
Potassium 5-ketogluconate	w	-	-	-	-	+
L-Rhamnose	+	-	-	-	-	-
Salicin	+	-	-	-	-	-
D-Xylose	-	-	-	+	-	-
**Biolog GEN III**						
N-Acetyl-D-glucosamine	-	+	+	+	+	-
N-Acetyl-*β*-D-mannosamine	+	-	-	+	-	-

Acetoacetic acid	+	+	+	-	+	-
Acetic acid	+	-	+	-	+	-
Aztreonam	+	+	+	+	+	-
L-Aspartic acid	+	+	-	-	-	-
Dextrin	+	-	-	+	-	-
D-Fructose	+	-	-	+	-	-
L-Fucose	+	+	+	+	-	-
D-Fructose 6-PO4	+	-	-	-	-	-
Glycyl-L-Proline	+	-	-	+	-	-
L-Glutamic acid	+	+	+	+	-	-
D-Galacturonic acid	+	-	-	+	-	-
L-Galactonic acid lactone	+	-	-	+	-	-
α-D-Glucose	-	+	+	+	+	-
α-D-Lactose	+	-	+	+	+	-
L-Lactic acid	+	-	-	-	-	-
D-Maltose	+	-	-	+	-	-
D-Mannose	+	+	+	+	-	-
Propionic acid	+	-	+	+	-	-
L-Serine	+	-	+	+	-	-
Sodium butyrate	+	+	+	+	-	-

Table S3. Cellular fatty acid compositions (%) of three novel species and reference strains in genus *Reichenbachiella*
Strains: 1, ABR2-5^{T}; 2, BKB1-1^{T}; 3, WSW4-B^{T}; 4, *R. agariperforans* KCTC 12369^{T}; 5, *R. faecimaris* KCTC 82811^{T}; 6, *R.versicolor* KCTC 82854^{T}

All data are from this study. Numbers indicate the percentages of the fatty acids. -, < 1%.

**Fatty acid (%)**	**1**	**2**	**3**	**4**	**5**	**6**
Saturated						
$C_{10:0}$	-	-	-	1.8	-	-
$C_{12:0}$	1.0	-	-	2.7	-	1.0
$C_{14:0}$	2.7	1.8	2.3	3.3	1.8	1.5
$C_{16:0}$	13.0	2.8	3.2	5.0	2.0	3.8
$C_{17:0}$	-	-	-	1.4	-	-
$C_{18:0}$	5.8	-	-	2.4	-	-
$C_{20:0}$	-	-	-	1.8	-	-
Unsaturated						
$C_{15:1}$ ω8c	-	-	-	-	3.6	-
$C_{16:1}$ ω5c	15.7	22.9	14.4	23.9	8.9	20.6
$C_{18:1}$ ω9c	6.4	-	-	1.0	-	-
Branched						
iso-$C_{14:0}$	-	-	1.7	-	2.1	4.0
iso-$C_{15:0}$	38.5	47.4	37.7	35.0	30.7	42.2
iso-$C_{15:1}$ F	-		9.7	-	14.4	-
anteiso-$C_{15:0}$	1.8	2.6	4.2	1.0	-	-
iso-$C_{16:0}$	1.3	3.4	2.5	1.0	5.9	-
Hydroxy						
$C_{15:0}$ 2-OH	-	-	-	-	2.1	-
$C_{16:0}$ 3-OH	1.1	1.3	1.0	-	-	3.0
iso-$C_{16:0}$ 3-OH	-	-	1.6	-	1.7	-
iso-$C_{17:0}$ 3-OH	2.3	3.5	2.5	3.0	1.7	2.5
Summed features*						
3	9.0	12.6	18.2	11.7	19.2	19.5

*Summed features are fatty acids that cannot be resolved reliably from another fatty acid using the chromatographic conditions chosen. The MIDI system groups these fatty acids together as one feature with a single percentage of the total. Summed feature 3 comprises $C_{16:1}\omega 7c$ or $C_{16:1}\omega 6c$.

Table S4. Genome features of three novel isolates: ABR2-5^T, BKB1-1^T, and WSW4-B4^T and three existing type strains in the genus *Reichenbachiella: R. agariperforans* DSM 26134^T, *R. faecimaris* DSM 26133^T, and *R. versicolor* DC003^T

Strains: 1, ABR2-5^T; 2, BKB1-1^T; 3, WSW4-B4^T; 4, *R. agariperforans* DSM 26134^T (Nedashkovskaya et al., 2005); 5, *R. faecimaris* DSM 26133^T (Cha et al., 2011); 6, *R. versicolor* DC003^T (Shi et al., 2018)

NA, not available

**Features**	**1**	**2**	**3**	**4**	**5**	**6**
Accession no.	GCA_025833875	GCA_025502585	GCA_025639805	GCA_900142205	GCA_900176375	GCA_003171675
Genome size (Mbp)	5.5	4.4	5.0	5.0	4.7	5.2
Completeness (%)	98.3	98.2	98.6	NA	NA	NA
G+C content (%)	42.0	42.1	41.8	43.4	39.8	37.1
Total genes	4619	3723	3855	4005	4004	4202
CDS	4568	3674	3806	3935	3929	4140
rRNAs (5S, 16S, 23S)	2, 2, 2	2, 2, 2	2, 2, 2	5, 1, 0	9, 3, 0	4, 1, 0
tRNAs	42	40	40	40	42	40
Pseudogenes	35	17	18	22	21	15

Table S5. ANI and dDDH values among three isolates and all three existing type strains in genus *Reichenbachiella,* using EzBioCloud server and Genome-to-Genome Distance calculator 3.0

Strains: 1, ABR2-5^{T}; 2, BKB1-1^{T}; 3, WSW4-B4^{T}; 4, *R. agariperforans* DSM 26134^{T}; 5, *R. faecimaris* DSM 26133^{T}; *R. versicolor* DC003^{T}

**Strains**	**1**	**2**	**3**	**4**	**5**	**6**	**Color scale**
**ANI value (%)**							
**1**	100	72.09	70.35	72.02	69.83	69.73	100
**2**	72.09	100	69.93	73.87	69.49	69.33	90
**3**	70.35	69.93	100	70.02	75.55	69.20	80
**4**	72.02	73.87	70.02	100	69.33	69.36	70
**5**	69.83	69.49	75.55	69.33	100	69.24	60
**6**	69.73	69.33	69.2	69.36	69.24	100	50
**dDDH value (%)**							
**1**	100	18.1	23.2	17.8	18.6	17.7	100
**2**	18.1	100	18.6	19.8	18.0	18.5	80
**3**	23.2	18.6	100	19.3	19.1	18.4	60
**4**	17.8	19.8	19.3	100	19.0	18.9	40
**5**	18.6	18.0	19.1	19.0	100	19.0	20
**6**	17.7	18.5	18.4	18.9	19.0	100	10

Table S6. Number of genes in the RAST subsystem categories of three novel strains, ABR2-5^T, BKB1-1^T, and WSW4-B4^T and three existing type strains in the genus *Reichenbachiella: R. agariperforans* DSM 26134^T, *R. faecimaris* DSM 26133^T, and *R. versicolor* DC003^T. The p- values for each novel strain were determined against the other two novel strains and three reference strains.

Strains: 1, ABR2-5^T; 2, BKB1-1^T; 3, WSW4-B4^T; 4, *R. agariperforans* DSM 26134^T; 5, *R. faecimaris* DSM 26133^T; *R. versicolor* DC003^T

RAST subsystem category distribution	1	2	3	4	5	6	*P* value		
							1	2	3
Cofactors, vitamins, prosthetic groups, pigments	120	121	141	121	137	118	0.38	0.21	0.33
cell wall and capsule	23	27	20	34	26	26	0.15	0.60	0.06
Virulence, disease and defense	36	31	30	30	29	20	0.01	0.43	0.04
Potassium metabolism	13	10	7	8	8	6	0.00	0.21	0.02
Miscellaneous	12	13	17	14	11	14	0.11	0.55	0.20
Phages, prophages, transposable elements, plasmids	0	0	0	0	1	1	0.14	0.14	0.14
Membranes transport	43	34	40	50	43	43	0.69	0.00	0.87
Iron acquisition and metabolism	4	0	1	4	2	7	0.34	0.02	0.60
RNA metabolism	43	31	37	35	34	39	0.00	0.01	0.02
Nucleosides and nucleotides	58	50	51	54	56	48	0.01	0.10	0.04
Protein metabolism	170	109	115	139	116	159	0.01	0.04	0.04
Cell division and cell cycle	3	3	4	3	4	3	0.14	0.14	0.33
Regulation and cell signaling	5	4	15	4	15	35	0.13	0.10	0.23
Secondary metabolism	4	5	5	5	6	4	0.02	0.58	0.08
DNA metabolism	59	55	50	58	47	54	0.02	0.53	0.08
Fatty acids, lipids, and isoprenoids	45	42	50	40	61	43	0.56	0.15	0.74
Nitrogen metabolism	21	16	18	16	11	16	0.01	0.80	0.02
Dormancy and sporulation	1	2	1	1	1	2	0.14	0.01	0.14
Respiration	48	47	34	45	26	38	0.04	0.07	0.12
Stress response	26	22	17	22	15	24	0.02	0.55	0.06
Metabolism of aromatic compounds	13	13	21	14	23	5	0.48	0.48	0.83
Amino acids and derivatives	222	216	235	211	253	232	0.33	0.08	0.51
Sulfur metabolism	25	22	42	20	7	37	0.92	0.49	0.55
Phosphorus metabolism	26	18	19	18	19	18	0.00	0.21	0.01
Carbohydrates	168	126	146	130	122	127	0.00	0.17	0.01

Table S7. Cas enzyme top 3 hits in the genome of strain ABR2-5^T according to the UniProt database

Query	Protein Names	Organism	Length(AA)	Identity(%)
**Cas1**	CRISPR-associated endonuclease Cas1, 3.1.-.-	*Reichenbachiella* sp. 5M10	305	86.9
	CRISPR-associated endonuclease Cas1, 3.1.-.-	*Tenuifilum thalassicum*	302	59.3
	CRISPR-associated endonuclease Cas1, 3.1.-.-	*Acidiluteibacter ferrifornacis*	300	58.4
**Cas2**	CRISPR-associated endoribonuclease Cas2, 3.1.-.-	*Reichenbachiella sp. 5M10*	112	89.3
	CRISPR-associated endoribonuclease Cas2, 3.1.-.-	*Daejeonella rubra*	109	75.9
	CRISPR-associated endoribonuclease Cas2, 3.1.-.-	*Mucilaginibacter paludis* DSM 18603	109	75.9
**Cas3**	RNA helicase	*Reichenbachiella* sp. 5M10	414	76.8
	ATP-dependent RNA helicase RhlE	*Reichenbachiella faecimaris*	412	70.7
	ATP-dependent helicase	*Pontibacter diazotrophicus*	421	68.3
**Cas9**	CRISPR-associated endonuclease Cas9 3.1.-.-	*Aquaticitalea lipolytica*	1,488	53.6
	CRISPR-associated endonuclease Cas9 3.1.-.-	*Kordia antarctica*	1,465	53.5
	CRISPR-associated endonuclease Cas9 3.1.-.-	*Winogradskyella epiphytica*	1,535	53

Table S8. Cas enzyme top 3 hits in the genome of strain BKB1-1^T according to the UniProt database

Query	Protein Names	Organism	Length(AA)	Identity(%)
**Cas1**	CRISPR-associated endonuclease Cas1, 3.1.-.-	*Reichenbachiella sp.* 5M10	305	88.8
	CRISPR-associated endonuclease Cas1, 3.1.-.-	*Tenuifilum thalassicum*	302	59
	CRISPR-associated endonuclease Cas1, 3.1.-.-	*Acidiluteibacter ferrifornacis*	300	58.7
**Cas2**	CRISPR-associated endoribonuclease Cas2, 3.1.-.-	*Reichenbachiella sp.* 5M10	112	83.2
	CRISPR-associated endoribonuclease Cas2, 3.1.-.-	*Daejeonella rubra*	109	72.3
	CRISPR-associated endoribonuclease Cas2, 3.1.-.-	*Chryseotalea sanaruensis*	109	69.3
**Cas3**	Ribonuclease Y, RNase Y, 3.1.-.-	*Reichenbachiella sp.* 5M10	520	93.7
	Ribonuclease Y, RNase Y, 3.1.-.-	*Reichenbachiella faecimaris*	521	86.9
	Ribonuclease Y, RNase Y, 3.1.-.-	*Marinoscillum furvescens* DSM 4134	519	80.4
**Cas9**	CRISPR-associated endonuclease Cas9, 3.1.-.-	*Acidiluteibacter ferrifornacis*	1,191	58.1
	CRISPR-associated endonuclease Cas9, 3.1.-.-	*Bacteroidota bacterium*	1,162	56.3
	CRISPR-associated endonuclease Cas9, 3.1.-.-	*Tenacibaculum jejuense*	1,295	48.3

Table S9. Cas enzyme top 3 hits in the genome of strain WSW4-B4^{T} according to the UniProt database

Query	Protein Names	Organism	Length(AA)	Identity(%)
**Cas1**	CRISPR-associated endonuclease Cas1, 3.1.-.-	*Reichenbachiella* sp. 5M10	305	86.9
	CRISPR-associated endonuclease Cas1, 3.2.-.-	*Thermophagus xiamenensis*	312	60.3
	CRISPR-associated endonuclease Cas1, 3.3.-.-	*Tenuifilum thalassicum*	302	58.8
**Cas2**	CRISPR-associated endoribonuclease Cas2, 3.1.-.-	*Reichenbachiella sp.* 5M10	112	84.2
	CRISPR-associated endoribonuclease Cas2, 3.1.-.-	*Daejeonella rubra*	109	75.2
	CRISPR-associated endoribonuclease Cas2, 3.1.-.-	*Pedobacter* sp. HMF7647	109	75.2
**Cas3**	Superfamily II DNA and RNA helicase	*Reichenbachiella faecimaris*	405	90.7
	ATP-dependent RNA helicase	*Reichenbachiella sp.* 5M10	407	77.8
	ATP-dependent RNA helicase	*Fulvivirga imtechensis* AK7	407	59.1
	RNA helicase	*Roseivirga spongicola*	404	56.2
**Cas9**	HNH Cas9-type domain-containing protein	*Reichenbachiella* sp. 5M10	1,373	97.6
	HNH Cas9-type domain-containing protein	*Flavobacterium sediminis*	1,371	55.4
	HNH Cas9-type domain-containing protein	*Hyunsoonleella pacifica*	1,405	53.9

Table S10. The percentage of CAZymes and the ratio of GHs/Mbp in the genome of three isolates and all three existing type strains in genus *Reichenbachiella*, based on dbCAN meta server

Strains: 1, ABR2-5^T; 2, BKB1-1^T; 3, WSW4-B4^T; 4, *R. agariperforans* DSM 26134^T; 5, *R. faecimaris* DSM 26133^T; *R. versicolor* DC003^T

GHs, glycoside hydrolases; GHs/Mbp, the number of GHs per mega base of genome

Strain	Genome Size (Mbp)	Number of Genes	Number of CAZyme	Percentage of CAZyme (%)	Number of GHs	Ratio of GHs/Mbp
1	5.5	4619	216	4.68	146	26.55
2	4.4	3723	97	2.61	41	9.32
3	5.0	3855	148	3.84	79	15.80
4	5.0	4005	182	4.54	109	21.80
5	4.7	4004	87	2.17	35	7.45
6	5.2	4202	102	2.43	59	11.35
